# Supplementary material for: Analysis of PPARγ Signaling Activity in Psoriasis
Source: Int J Mol Sci. 2021 Aug 10;22(16):8603. doi: 10.3390/ijms22168603 (PMC8395241; doi:10.3390/ijms22168603)
Supplement: Supplementary file 1 [file ijms-22-08603-s001.zip › Supplemental materials_Analysis of PPARg signaling activity in psoriasis/Pathway models/Models images and html files/Model of PPARG related pathways in psoriasis (short version)/11037.html]

STK11


# Protein STK11

|  |  |
| --- | --- |
| URN | urn:agi-llid:6794 |
| Total Entities | 0 |
| Connectivity | 2748 |
| Name | STK11 |
| Description | serine/threonine kinase 11 |
| Notes | This gene, which encodes a member of the serine/threonine kinase family, regulates cell polarity and functions as a tumor suppressor. Mutations in this gene have been associated with Peutz-Jeghers syndrome, an autosomal dominant disorder characterized by the growth of polyps in the gastrointestinal tract, pigmented macules on the skin and mouth, and other neoplasms. Alternate transcriptional splice variants of this gene have been observed but have not been thoroughly characterized. [provided by RefSeq, Jul 2008] |
| Primary Cell Localization | Cytoplasm |
| Class | Protein kinase |

---

|  |  |
| --- | --- |
| Pathway | Proteins Involved in Stem Cell Exhaustion in Aging |
|  | DNA Persistent Repair Inhibits mTOR Signaling |
|  | mTOR Signaling |
|  | Hypoxia Inhibits mTOR Signaling |
|  | SIRT3 Signaling in Aging |
|  | mTOR Signaling Activation by Taste Sensor Receptors (Rodent Model) |
|  | Adipokines and Cytokines in Insulin Resistance in Skeletal Muscles |
|  | Adiponectin in Insulin Resistance Prevention |
|  | Insulin Resistance in Myocytes Induced by Obesity |
|  | Insulin Resistance in Hepatocytes |
|  | Peutz-Jeghers Syndrome |
|  | Proteins Involved in Peutz-Jeghers Syndrome |
|  | Proteins Involved in Hepatocellular Carcinoma |
|  | Proteins Involved in Pancreatic Cancer |
|  | Pancreatic Ductal Carcinoma |
|  | STK11 Signaling in Pancreatic Neoplasms |
|  | Proteins Involved in Polycystic Ovary Syndrome |
|  | Ketogenic Diet Mechanism (Hypothesis) |
|  | Proteins Involved in Polycystic Kidney Disease |
|  | Podocyte Dysfunction in Glomerulonephritis |
|  | Genes with Mutations in Cancer-Associated Evading of Growth Suppressors |
|  | PI3K/AKT/MTOR Signaling Activation by Blocking of Tumor Suppressors |
|  | Proteins with Altered Expression in Cancer-Associated Evading of Growth Suppressors |
|  | Metabolic Effects of Oncogenes and Tumor Suppressor in Cancer Cells |
|  | Genes with Mutations in Cancer Metabolic Reprogramming |
|  | Proteins with Altered Expression in Cancer Metabolic Reprogramming |
|  | Lysophosphatidic Acid/LPARs Signaling |
|  | AMPK Signaling |
|  | mTOR Signaling |
|  | Model of PPARG signaling in psoriasis |
|  | Model of PPARG related pathways in psoriasis (short version) |
|  | 4\_3\_Insulin resistance: FFA-related insulin resistance |
|  | Model of PPARG signaling in psoriais (tested) |

---

|  |  |
| --- | --- |
| Group | Genes with Mutations Associated with Peutz-Jeghers Syndrome |
|  | Genes with Mutations Associated with Pancreatic Cancer |
|  | Proteins Involved in Cowden Syndrome |
|  | Genes with Mutations Associated with Endometrial Cancer |
|  | Genes with Mutations Associated with Ovarian Cancer |

---

|  |  |
| --- | --- |
| MedScan ID | 6794 |

---

|  |  |
| --- | --- |
| LocusLink ID | 6794 |
|  | 20869 |
|  | 314621 |

---

|  |  |
| --- | --- |
| Alias | LKB1 |
|  | OTTMUSP00000043767 |
|  | R75140 |
|  | serine/threonine kinase 11 (Peutz-Jeghers syndrome) |
|  | AA408040 |
|  | OTTMUSP00000020599 |
|  | polarization-related protein LKB1 |
|  | renal carcinoma antigen NY-REN-19 |
|  | LKB1 kinase |
|  | protein kinase LKB1 |
|  | serine/threonine-protein kinase STK11 |
|  | LKB1 short isoform |
|  | Serine/threonine- protein kinase LKB1 |
|  | liver kinase B1 homolog |
|  | serine/threonine kinase 11 |
|  | LKB I |
|  | hLKB1 |
|  | liver kinase B1 |
|  | mLKB1 |
|  | Serine/threonine- protein kinase LKB I |
|  | OTTMUSP00000020600 |
|  | STK11 |
|  | PJS |
|  | serine/threonine-protein kinase 11 |
|  | serine/threonine-protein kinase LKB1 |
|  | Par-4 |

---

|  |  |
| --- | --- |
| EC Number | 2.7.11.1 |

---

|  |  |
| --- | --- |
| GO ID | 0005524 |
|  | 0030275 |
|  | 0000287 |
|  | 0002039 |
|  | 0030295 |
|  | 0004674 |
|  | 0051645 |
|  | 0050852 |
|  | 0032147 |
|  | 0043276 |
|  | 0006914 |
|  | 0007409 |
|  | 0060070 |
|  | 0007050 |
|  | 0006974 |
|  | 0071493 |
|  | 0097484 |
|  | 0030010 |
|  | 0042593 |
|  | 0072332 |
|  | 1904262 |
|  | 0090090 |
|  | 0030308 |
|  | 0008285 |
|  | 0120163 |
|  | 0060770 |
|  | 0010508 |
|  | 0050772 |
|  | 1900182 |
|  | 0030511 |
|  | 0045059 |
|  | 0046777 |
|  | 0006470 |
|  | 0006468 |
|  | 0001558 |
|  | 0048814 |
|  | 0051896 |
|  | 1901796 |
|  | 0010212 |
|  | 0007283 |
|  | 0001894 |
|  | 0001944 |
|  | 0005737 |
|  | 0005829 |
|  | 0070062 |
|  | 0016020 |
|  | 0005739 |
|  | 0005654 |
|  | 0005634 |
|  | 0016301 |
|  | 0046872 |
|  | 0000166 |
|  | 0004672 |
|  | 0044877 |
|  | 0016740 |
|  | 0036399 |
|  | 0006915 |
|  | 0007049 |
|  | 0030154 |
|  | 0051055 |
|  | 0016310 |
|  | 0045722 |
|  | 0050731 |
|  | 0071902 |
|  | 0051291 |
|  | 0030111 |
|  | 0033993 |
|  | 0007286 |
|  | 0036398 |
|  | 0032991 |
|  | 0033762 |
|  | 0043434 |
|  | 0032403 |
|  | 0043234 |
|  | 0006112 |
|  | 0008286 |
|  | 0042304 |
|  | 0044281 |
|  | 0016772 |
|  | 0045860 |

---

|  |  |
| --- | --- |
| KEGG ID | hsa:6794 |
|  | mmu:20869 |

---

|  |  |
| --- | --- |
| Organism | Homo sapiens {Organism urn:agi-taxid:9606} |
|  | Mus musculus {Organism urn:agi-taxid:10090} |
|  | Rattus norvegicus {Organism urn:agi-taxid:10116} |
|  | Homo sapiens |
|  | Mus musculus |
|  | Rattus norvegicus |

---

|  |  |
| --- | --- |
| Mouse chromosome position | 10 |

---

|  |  |
| --- | --- |
| OMIM ID | 602216 |
|  | 260350 |
|  | 273300 |
|  | 175200 |

---

|  |  |
| --- | --- |
| Rat chromosome position | 7q11 |

---

|  |  |
| --- | --- |
| Hugo ID | 11389 |
|  | HGNC:11389 |

---

|  |  |
| --- | --- |
| Human chromosome position | 19p13.3 |

---

|  |  |
| --- | --- |
| Swiss-Prot Accession | A0A0S2Z4D1 |
|  | Q15831 |
|  | Q15831.1 |
|  | Q9WTK7 |
|  | Q3V4A1 |
|  | Q9WTK7.1 |
|  | A0A0H2UI02 |
|  | D4AE59.1 |
|  | B2RBX7 |
|  | E7EW76 |
|  | B3VBP0 |
|  | Q3TAE0 |
|  | D4AE59 |

---

|  |  |
| --- | --- |
| GenBank ID | NC\_000019 |
|  | NM\_000455 |
|  | NP\_000446 |
|  | NG\_007460 |
|  | AC004221 |
|  | AAF97257 |
|  | AC011544 |
|  | AH005839 |
|  | AAB97833 |
|  | AH006100 |
|  | AAC15742 |
|  | CH471139 |
|  | EAW69538 |
|  | EAW69539 |
|  | EAW69540 |
|  | AB209553 |
|  | BAD92790 |
|  | AF035625 |
|  | AAC39527 |
|  | AF217978 |
|  | AAG17221 |
|  | AJ844634 |
|  | CAH59749 |
|  | AK128518 |
|  | AK314858 |
|  | BAG37374 |
|  | BC007981 |
|  | AAH07981 |
|  | BC019334 |
|  | AAH19334 |
|  | BM993274 |
|  | BQ222023 |
|  | BU174122 |
|  | BU739451 |
|  | EF644172 |
|  | ABR45718 |
|  | GU573916 |
|  | ADD71158 |
|  | KU178339 |
|  | ALQ33797 |
|  | KU178340 |
|  | ALQ33798 |
|  | U63333 |
|  | AAB05809 |
|  | Q15831 |
|  | NC\_000076 |
|  | XM\_006513443 |
|  | XP\_006513506 |
|  | XM\_006513441 |
|  | XP\_006513504 |
|  | XM\_006513442 |
|  | XP\_006513505 |
|  | NR\_126043 |
|  | NM\_011492 |
|  | NP\_035622 |
|  | NM\_001301854 |
|  | NP\_001288783 |
|  | NM\_001301853 |
|  | NP\_001288782 |
|  | XM\_006513439 |
|  | XP\_006513502 |
|  | AB026255 |
|  | BAA86229 |
|  | AC159999 |
|  | AF145697 |
|  | AAD55369 |
|  | AH008186 |
|  | AAD55368 |
|  | CH466553 |
|  | EDL31600 |
|  | EDL31601 |
|  | AB015801 |
|  | BAA76749 |
|  | AF129870 |
|  | AAD22100 |
|  | AF145287 |
|  | AAD31044 |
|  | AF151711 |
|  | AAF21370 |
|  | AK004499 |
|  | BAE43183 |
|  | AK147802 |
|  | AK171909 |
|  | BAE42728 |
|  | AK172385 |
|  | BAE42977 |
|  | AK172528 |
|  | BAE43050 |
|  | BC052379 |
|  | AAH52379 |
|  | BE951985 |
|  | BF468348 |
|  | CD565465 |
|  | CJ229363 |
|  | CO424479 |
|  | EU730638 |
|  | ACE73833 |
|  | KY779728 |
|  | AVC68843 |
|  | Q9WTK7 |
|  | NC\_005106 |
|  | NM\_001108069 |
|  | NP\_001101539 |
|  | XM\_008765096 |
|  | XP\_008763318 |
|  | XM\_006240910 |
|  | XP\_006240972 |
|  | AC\_000075 |
|  | AAHX01047516 |
|  | AC141331 |
|  | CH474029 |
|  | EDL89333 |
|  | EDL89334 |
|  | D4AE59 |
|  | XM\_011528209 |
|  | XP\_011526511 |
|  | XM\_005259617 |
|  | XP\_005259674 |
|  | XR\_936204 |
|  | XM\_005259618 |
|  | XP\_005259675 |
|  | NC\_018930 |
|  | AF032984 |
|  | AF032985 |
|  | AF032986 |
|  | AF055320 |
|  | AF055321 |
|  | AF055322 |
|  | AF055323 |
|  | AF055324 |
|  | AF055325 |
|  | AF055326 |
|  | AF055327 |
|  | AMYH02035142 |
|  | DQ893232 |
|  | ABM84158 |
|  | DQ896561 |
|  | ABM87560 |
|  | AC\_000032 |
|  | AAHY01086929 |
|  | AF145288 |
|  | AF145289 |
|  | AF145290 |
|  | AF145291 |
|  | AF145292 |
|  | AF145293 |
|  | AF145294 |
|  | AF145295 |
|  | AF145296 |
|  | AF145297 |
|  | AC\_000151 |
|  | ABBA01002630 |
|  | ABBA01002631 |
|  | XR\_380381 |
|  | XM\_006513440 |
|  | XP\_006513503 |

---

|  |  |
| --- | --- |
| Swiss-Prot ID | STK11\_HUMAN |
|  | STK11\_MOUSE |

---

|  |  |
| --- | --- |
| Cell Localization | Nucleus |
|  | Cytoplasm |
|  | Membrane |
|  | Mitochondrion |

---

|  |  |
| --- | --- |
| Ensembl ID | ENSG00000118046 |
|  | ENSP00000324856.6 |
|  | ENST00000326873.11 |
|  | ENSMUSG00000003068 |
|  | ENSMUSP00000150488.1 |
|  | ENSMUST00000213772.1 |
|  | ENSMUSP00000101009.1 |
|  | ENSMUST00000105370.7 |
|  | ENSMUSP00000003152.7 |
|  | ENSMUST00000003152.13 |
|  | ENSRNOG00000014287 |
|  | ENSRNOP00000057414.3 |
|  | ENSRNOT00000060683.3 |
|  | ENSP00000324856 |
|  | ENST00000326873 |
|  | ENSMUSP00000114195 |
|  | ENSMUST00000144883 |
|  | ENSMUSP00000101009 |
|  | ENSMUST00000105370 |
|  | ENSMUSP00000003152 |
|  | ENSMUST00000003152 |
|  | ENSRNOP00000057414 |
|  | ENSRNOT00000060683 |
|  | ENSP00000467240 |
|  | ENST00000586243 |

---

|  |  |
| --- | --- |
| MGI ID | MGI:1341870 |

---

|  |  |
| --- | --- |
| RGD ID | 1308653 |

---

|  |  |
| --- | --- |
| Unigene ID | Hs.515005 |
|  | Mm.44231 |
|  | Rn.12052 |

---

|  |  |
| --- | --- |
| Homologene ID | 393 |

---

|  |  |
| --- | --- |
| Shape | Sickle-vertex |

---
